# The Advances and Challenges of NK Cell-Based Cancer Immunotherapy

**DOI:** 10.3390/curroncol28020105

**Published:** 2021-02-26

**Authors:** Synat Kang, Xuefeng Gao, Li Zhang, Erna Yang, Yonghui Li, Li Yu

**Affiliations:** 1 Department of Hematology and Oncology, Shenzhen Key Laboratory of Precision Medicine for Hematological Malignancies, International Cancer Center, Shenzhen University General Hospital, Shenzhen University Health Science Center, Shenzhen University, Xueyuan AVE 1098, Nanshan District, Shenzhen 518000, Guangdong, China; kang_synat@szu.edu.cn (S.K.); lizhang2019@szu.edu.cn (L.Z.); yangerna@szu.edu.cn (E.Y.); yonghuili@szu.edu.cn (Y.L.); 2 Central Laboratory, Shenzhen University General Hospital, Xueyuan AVE 1098, Nanshan District, Shenzhen 518000, Guangdong, China

**Keywords:** natural killer cells, cancer immunotherapy, T cell receptor, TCR-NK

## Abstract

Natural killer (NK) cells can be widely applied for cancer immunotherapy due to their ability to lyse tumor targets without prior sensitization or human leukocyte antigens-matching. Several NK-based therapeutic approaches have been attempted in clinical practice, but their efficacy is not sufficient to suppress tumor development mainly because of lacking specificity. To this end, the engineering of NK cells with T cell receptor along with CD3 subunits (TCR-NK) has been developed to increase the reactivity and recognition specificity of NK cells toward tumor cells. Here, we review recent advances in redirecting NK cells for cancer immunotherapy and discuss the major challenges and future explorations for their clinical applications.

## 1. Introduction

Natural killer (NK) cells are known as the non-specific immune system that screen cell surfaces of autologous cells for abnormal expression of MHC class I molecules and cell stress marker [1]. NK cells were first identified in mice in 1975 as a subgroup of lymphocytes endowed with the capacity to eliminate cancerous cells without presenting the MHC class I molecule [2]. Since then, NK cells became a main ideology in terms of their unspecific killer machines and vital catalyzers of adaptive T-cell responses.

NK cells have been investigated clinically in several immunotherapeutic strategies for various cancers. Evidence has shown high efficacy of NK cells mediating direct killing of freshly isolated human tumor cells from hematopoietic and solid tumors [3,4]. Moreover, adoptive cell therapy (ACT) treatment using alloreactivity NK cells was safe and effective for patients with metastatic melanoma, colon carcinoma, refractory Hodgkin’s disease, and recurrent acute myeloid leukemia (AML) [5,6,7,8]. However, not all tumors appeared to respond to this type of ACT therapy. In some cases, tumor cells can evade NK cell clearance due to lacking antigen specificity. Gene-modified NK cells with chimeric antigen receptor (CAR) have been shown to enhance the effector cell function and antigen-specificity against several tumor targets, including anti-CD19 CAR-NK for targeting and chronic lymphocytic leukemia (CLL) [9] and anti-CD138 CAR-NK for targeting multiple myeloma patients [10]. Although the therapeutic effectiveness and safety of CAR-NK cell therapy have been reported, the usage of CAR-NK cell-based therapy is still faced with several obstacles, including low efficiency of CAR-transduction, limited cell expansion, and lack of available targets [11]. TCR-transduced T cells (TCR-T) have been used in clinical trials against a wide variety of tumor antigens, particularly the cancer-testis antigens (CTA) [12,13,14]. Recently, two reports tested the efficacy of TCR in combining with NK cell lines for targeting malignant cancer [15,16]. However, a concern of TCR gene transfer redirecting T cells is the mispairing of introduced TCR chains with endogenous chains [17]. Herein, we discuss the major challenges and future directions for the clinical application of NK cells.

## 2. Interplay between NK Cells and Cancer Cells

**Figure 1:**
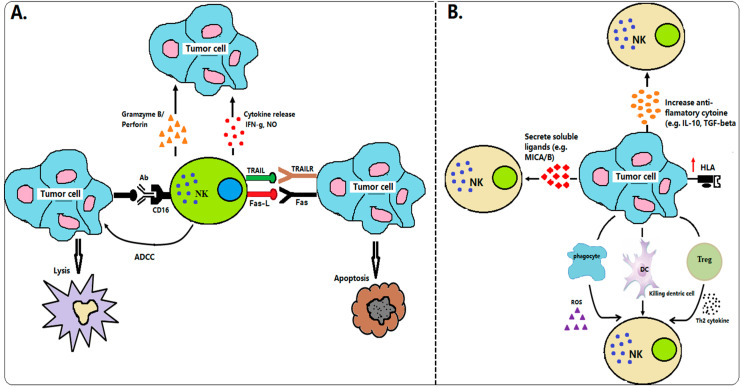
Interactions between NK cells and cancer cells in immunosurveillance. (**A**) Mechanism of NK cells in tumor immunosurveillance. NK cells can identify the tumor cells by stress or danger signals. Upon stimulation, NK cells directly killed tumor cells through many tactics, including the release of cytokine productions (e.g., IFN-g, NO), cytoplasmic granule release (e.g., granzyme B, perforin), death receptor-induced apoptosis (e.g., Fas-L, TRAIL), and ADCC. (**B**) Mechanism of tumor cells invading NK cells. Tumor cells defend themselves from NK cell attack through several techniques, including secretion soluble ligand of NK cell receptors (e.g., MICA/B), upregulation of HLA molecules, secretion immunosuppressive factor products (e.g., TGF-β or IL-10), and activation of Treg or phagocyte-derived inhibitory cytokines (ROS).

NK cell population is about 10–15% in a whole of human peripheral blood lymphocytes and is regarded as a natural killer as they have cytotoxic properties against tumor cells without any prior priming (e.g., as required by CD8 T cells) [18]. They have been considered of great importance in terms of immunosurveillance, as they recognize and kill different types of target cells, such as virus-infected cells and malignant cells. The majority (~90%) CD56dim of the total NK cell population in peripheral blood expresses high levels of FcγRIII (CD16), whereas a small population (~10%) CD56bright of NK cells are mostly involved in the production of cytokines [18,19]. NK cells do not undergo antigen-specific receptor rearrangement as T and B lymphocytes, instead of the functional activities of NK cells to lyse the target process through their germline-encoded immunoreceptors [20]. NK cells protect the host from infectious or cancers by expressing activating and inhibitory receptors. Activated NK cells can recognize and eliminate the target cells by the balance of the signaling derived from inhibitory receptors (e.g., KIRS or NKG2A) and activating receptors (e.g., NCRs or NKG2D) [21,22]. Moreover, NK cells are involved in the regulation of the immune response by the expression of different chemokines and chemokine receptors such as CCL4, CXCL8, and CXCR3 [23,24]. Klas Karre, the first person, as part of his doctoral thesis, proposed that NK cytolysis of a target cell could be triggered by a decrease or absence of host major histocompatibility class I molecules (MHC-I) on the surface of a target cell [25]. This hypothesis was then confirmed by several other groups [26,27,28]. NK cells are inactivated when their inhibitory receptors identify the self-MHC class I molecule, and thus they can protect the cells from host cell attacking [29]. In cancer patients who are low/deficient of MHC-class I or bear “altered-self” stress-inducible proteins can be targeted by NK cell killing and cytotoxicity "the missing self-hypothesis" of Klas Karre [25]. NK cells kill tumor cells through several mechanisms, including the release of cytoplasmic granules containing perforin and granzyme, secretion of immunoregulatory cytokines such as nitric oxide (NO), and expression of other TNF-family members such as Fas-L or TRAIL (Figure 1A). However, tumor cells can evade host immune response via multiple strategies, including weak immunogenicity of target antigens and the creation of an immune-suppressive tumor environment (Figure 1B).

## 3. NK Cells in Cancer Immunotherapy

The ability to recognize and lyse tumor cells via a variety of recognition receptors make NK cells a candidate for cancer immunotherapy. Several strategies of using NK cells have been attempted for clinical practice in cancer immunotherapy (Table 1) [30,31,32]. Some of these approaches include isolation of immune cells and expansion cell with cytokines (e.g., autologous and allogeneic NK cells, and NK cell lines), and other strategies involved genetically modified immune cells with specific-target genes (e.g., CAR-NK and TCR-NK). Despite some successes, a large proportion of patients failed to respond to NK cell-based immunotherapy.

### 3.1. Autologous NK Cell Therapy

Early trials of autologous NK cell therapy from a leukapheresis product have demonstrated high potency against several advanced metastatic cancers [59,60]. In one clinical trial (UMIN000007527), autologous NK cell therapy was very effective in patients with advanced digestive, colon, and lung cancer, and adverse event was not observed [61]. However, some other studies showed that these autologous NK cells failed to demonstrate clinical responses or efficacy [34,62]. This failure was mainly due to the inhibitory receptors on autologous NK cells matched self MHC class I presented on cancer cells, which subsequently suppressed the activation of NK cells [63,64]. In addition, autologous NK cells derived from cancer patients were usually in an immunosuppression state with impaired functions, making these cells difficult to demonstrate antitumor functionality.

Previous studies proposed that NK cell antitumor activity could be enhanced by the systemic administration of cytokines. Systemic cytokine administration in combination with NK cells was a major strategy for adoptive cancer immunotherapy [65,66,67,68]. Optimization by using adoptive transfer of ex vivo IL-2 activated NK cells showing better outcomes than the systemic administration of IL-2 [61]. Although cytokine IL-2-activated autologous NK cells can boost NK cell activation and exhibit high efficacy against malignant tumors, existing studies showed that this strategy had limited success [35]. Lymphokine-activated killer (LAK) cells in combination with IL-2 displayed upregulated adhesion molecules and activating receptors and secreted inflammatory cytokines to adhere and lyse metastatic renal cell carcinoma (RCC) [69]. However, after adoptively transferring LAK cells, patients received toxic side effects of vascular leak syndrome due to a high level of IL-2 [70]. Another study demonstrated that transferring of ex vivo expanded autologous NK cells with IL-15 was potent regression in non-Hodgkin lymphoma (NHL) patients [71]. However, extremely high doses of IL-15 were associated with cytokine release syndrome in the patients with advanced acute myeloid leukemia (AML) [72] and dose-limiting toxicities in the patient malignant melanoma or renal cell cancer [73]. In contrast to autologous NK cell therapy, stimulation human haploidentical NK cells with IL-15 cytokine showed complete hematologic remission in poor-prognosis AML patients [5].

### 3.2. Allogeneic NK Cell Therapy

Unlike T cells, adaptive transferring of NK cells does not promote GVHD, and therefore life-threatening toxicity of patients of allogeneic donor NK cell administration is negligible. KIR-ligand mismatches have been trialed in patients with AML and showed increasing overall survival, better engraftment, and a reduced incidence of GVHD after receiving haploidentical T cell-depleted allogeneic stem cell transplantation [74]. Allogeneic NK cells with KIR mismatch offer greater cytotoxicity than autologous NK cells and can be effective at controlling AML relapse [5,75]. Moreover, clinical evidence also agrees with the therapeutic effect of allogeneic NK cells in controlling human malignancies, including high-risk leukemia, renal cell carcinoma, and others [5,46,76,77]. Allogeneic NK cells expanded with IL-15 and hydrocortisone are being tested for the treatment of non-small cell lung carcinoma in a Phase I safety clinical trial [40]. The result of other Phase I clinical trials also showed high efficacy and safety of allogeneic NK cells in combing with interleukin 21 (mbIL-21) expansions for targeting the advanced myeloid malignancies [39]. The advantages of allogeneic NK cell transfusion include that these cells are cultivated or well-educated in healthy hosts and have high-efficiency killing cancer cells. However, using KIR mismatched allogeneic NK cells sometimes created immune-mediated rejection due to MHC mismatch. The study of KIR/HLA genotype has been demonstrated that a large population of patients was associated with acute rejection after kidney transplantation because of KIR/HLA polymorphism [42]. In Phase II clinical trials (NCT00703820), adaptive allogeneic NK cells from KIR–HLA-mismatched donors failed to respond to the intermediate- or high-risk AML. The failure could be the outcome of insufficient numbers and limited persistence of alloreactive donor NK cells [44]. The major limitation of using allogeneic NK cells in therapy is the problem of yielding an adequate cell number. Therefore, the optimization ex vivo expansion and activation strategies remain a major focus [44]. In addition, allogeneic NK cells from donors may be risky for cancer immunotherapy due to unpredictable T or B lymphocyte presenting.

### 3.3. NK Cell Lines

The NK cell lines, such as NK-92 or NK-92MI, NKL, NKG, KHYG-1, and YT, have been generated from malignant NK cell clones [78]. NK cell lines are purity NK cells, which are unlimited cell expansion and proliferation, and can obtain a sufficient amount of the cells in a short period, and hence it can transplant to patients on a regular schedule. Only NK-92 has shown a high antitumor activity in several types of tumor and has worked well in pre-clinical development [78,79,80]. NK-92 was generated from a non-Hodgkin Lymphoma patient and could kill hematopoietic cancers in vitro [81]. NK-92 cells expressed most of the activating receptor but rarely expressed inhibitory killer cell immunoglobulin-like receptors (KIRs) except for KIR2DL4, which can hamper NK cell activation and killing the target cells [80]. Alternatively, NK-92MI cells have similar characteristics of activated NK cells as their origin NK-92 cells and obtain the gene expression by transduction with human IL-2 cDNA [82]. 

NK-92 has received US FDA approval for testing in patients with solid tumors, and the non-modified NK-92 has completed Phase I trials [45,46,47,83]. For minor risk factors, cell lines need to irradiate priority before transfusion to patients. Thus, NK-92 cells are unable to expand severely in vivo, which can decrease their efficacy to target the tumor. Several Phase I clinical trials data have shown the safety and tolerability of NK-92 cells, however, the results are still unsatisfied with their clinical benefit [47,48]. For example, no antitumor response of the adoptive transfer of NK-92 cells was observed in all patients with refractory/relapsed AML of Phase I clinical trial (NCT00900809) [45].

### 3.4. Antibody-Based NK Cell Therapy

Antibody-based drugs are widely used in cancer immunotherapy. The activating type IIIa Fc receptor (FcγRIIIA) or CD16 expression by NK cells enables them to bind to antibody-coated targets initiating ADCC pathway that eventually results in the elimination of target cells [84,85,86]. The roles of ADCC in the efficacy of therapeutic antitumor monoclonal antibodies have shown in the patients with non-Hodgkin’s lymphoma (NHL) for rituximab (anti-CD20) and metastatic breast and gastric carcinoma for herceptin (anti-HER2) [87,88]. Currently, the use of margetuximab (anti-HER2) plus pembrolizumab (anti-PD1 checkpoint blockade) has been demonstrated highly effective for treating patients of HER2-positive gastro-oesophageal adenocarcinoma [89]. Moreover, in the clinical trial (NCT03248492), trastuzumab deruxtecan (antibody-drug conjugate) has been demonstrated high efficacy, prolong progression-free survival and safety in the patients with Her-2 positive metastatic breast cancer [90]. Alemtuzumab (anti-CD52), which activates NK cell effectors, has been shown to have high-efficiency in patients with B-CLL, and GVHD post-HSCT [91,92]. Dinutuximab is a product of human-mouse chimeric mAb (ch14.18 mAb), which can mediate ADCC through NK cell receptors. This product has demonstrated high efficacy against GD2-positive neuroblastoma cells in vitro and melanoma cells in vivo [93,94,95]. Use of cetuximab in combination with irradiated high affinity (ha) NK cells has shown highly lysed chordoma cancer [53]. Daratumumab (a mAb against CD38)-induced NK cells via ADCC mechanism have demonstrated effective elimination of multiple myeloma (MM) in pre-clinical and clinical studies [49,96,97]. In clinical trials (NCT03158688 and NCT01998971), Daratumumab plus carfilzomib and dexamethasone have shown effective clinical response and prolong progression-free survival (PFS) in patients with relapsed or refractory MM [97,98]. Moreover, monoclonal antibodies (zalutumumab and necitumumab), anti-EGFR mAb, have been shown efficacy for treating patients with squamous cell carcinoma of the head and neck and have been approved by the FDA [99,100]. In MHC-I expressing tumor cells, the effector functions of autologous NK cells are often inhibited by KIR. Therefore, strategies to block KIR expression using antibodies have been developed to potentiate NK cell cytotoxicity. The anti-KIR (IPH2101) mAb is being tested in Phase I clinical trial (EUDRACT: 2005-005298-31). Interestingly, IPH2101 mAb can block KIR-mediated inhibition of NK cells to enhance cytotoxicity against AML blasts [51] and multiple myeloma [52]. Moreover, several clinical trials of using therapeutic mAb in combination with NK cells for cancer treatments have been reported in elsewhere reviews [101,102,103,104,105,106,107]. Although the therapeutic monoclonal antibodies have shown a promising therapeutic efficacy for many cancer types, dose optimization and optimal management of toxicity are still needed to be determined before infusing them into patients [54]. 

### 3.5. Genetic Modification of CAR-NK Cells

The cytokine gene transfer approaches, including interleukins and stem cell factor (SCF), have been shown to induce NK cell proliferation and increases survival capacity in vivo functional activity [108,109]. However, these strategies are still limited due to NK cell specificity. Genetic manipulation of chimeric antigen receptors (CAR) redirecting NK cells (CAR-NK) could be an effective approach to mediate specific NK cell antitumor effects against different targets. This technique is based on the transduction of NK cells to target tumor cells by gene transfer of CAR-specific receptors through recombinant a single-chain variable fragment receptor (Fv) specific of a tumor-associated specific antigen to downstream intracellular signaling machinery [110]. 

The use of primary CAR-NK and CAR-NK lines in pre-clinical and clinical trials for targeting specific tumor have been reported in elsewhere reviews [56,111,112]. Pre-clinical studies in hematological tumors showed high specificity and cytotoxicity toward the target cells [55,113,114,115,116,117,118,119,120,121,122,123]. For example, the transduction of cord blood (CB)-derived NK cells with a retroviral vector of CAR-CD19 containing IL-15 and suicide caspase-9-based suicide (iC9/CAR.19/IL-15 CB-NK cells) have demonstrated to be efficient in killing CD19-expressing cell lines and primary leukemia cells in vitro, xenograft model, and in the clinical trial (NCT03056339) [9,124]. Moreover, the CAR-NK-based showed a potent antitumor effect against several solid tumors in pre-clinical studies [125,126,127].

So far, only a few clinical trial studies of CAR-NK for targeting hematological or solid tumors have been registered on ClinicalTrials.gov (Table 2). Two clinical trials were the origin of NK cell lines, including BCMA (NCT03940833) for targeting malignant multiple myeloma (MM) and CD33 (NCT02944162) for targeting AML. The other trials were the origin from human primary NK cells, including ROBO1 for targeting solid tumor (NCT03940820), Mesothelin for targeting epithelial ovarian cancer (NCT03692637), PSMA for targeting prostate cancer (NCT03692663), CD22 for targeting refractory B cell lymphoma (NCT03692767), CD19 for targeting refractory B cell lymphoma (NCT03690310), CD19/CD22 for targeting metastatic solid tumors (NCT03824964), NKG2D for targeting relapsed and refractory B cell lymphoma (NCT03415100), and CD19/iCasp9/IL15 for targeting B cell non-Hodgkin lymphoma (NHL) (NCT03579927).

Although CAR-NK therapy has successfully entered into clinical trials, its clinical application is often associated with some challenges. First, the obstacle of the clinical application of primary CAR-NK is the limitation of cell expansion for flexible schedule transfusion [56]. Stimulation of NK cells with feeder cells has been shown to expand the number of NK cells. However, the final NK cell products are possible containing the feeder cells, which could be a potential risk in clinical usage [128]. Second, NK cells are hard to achieve high transduction efficiency than other cells of the hematopoietic system [57]. The transfection efficiency of peripheral blood (PB) and cord blood (CB) with mRNA showed low efficacy: less than 10% (PB and CB), while lentiviral transduction showed ~8–16% in PB and ~12–73% in CB [115]. Although electroporation mRNA transfection has seen better efficiency in CB, the expression of CAR molecule is unstable and losses expression within a few days, which are the barrier for ACT [118]. Third, the freeze-thaw process of NK cells was associated with loss of functional activity [58]. There have been reported the severe loss of cytolytic function of cryopreservation IL-15-activated NK cells (92–98% reduction), followed by overnight culturing the cell without cytokine IL-15 [58]. Hence, similar to CAR-NK immunotherapy, genetically modified NK cells using TCR molecules could be a better option for cancer treatment.

### 3.6. TCR Transduced NK Cells in Cancer Immunotherapy

Recent technology of genetically modified NK cells could be used to study different pathways involved in NK cell tumor targeting and to improve their tumor cytotoxicity [129]. NK cells lack the expression of the TCR complex subunits (except CD3ζ); however, they do express all necessary molecules for downstream signaling [130]. The possibility to apply such gene-transfer strategies to NK cells appears fascinating. It is possible to give extra tumor-antigen specificity to NK cell, which is already MHC-independent antitumor activity. TCR, as a natural antigen receptor, is effectively recognizing several proteins of the tumor. Therefore, it can redirect to cells such as NK cells against the tumors. The wild-type TCR can generate from the low avidity of T cells, and its affinity can enhance by using the phage display library [13,131]. Techniques used to transfer the gene redirecting NK cells, including viral transduction (retroviral/lentiviral) and transfection (DNA/RNA electroporation) [129]. Lentiviral transduction was a potential approach and safe in the clinic and showed efficient transduction and stable integration of transgenes [132,133]. Similar to CAR-NK products, TCR-NK products also could be prepared for clinical use. It has been known that loss or downregulation of HLA Ia in human tumors usually facilitates immune evasion from CD8+ CTL-mediated killing [134]. However, the absence or downregulation of HLA class Ia can promote the expression of new tumor peptides presented by HLA-E, therefore activating unconventional CD8+ T cells through HLA-E–peptide–specific recognition by TCRs. This feature endows an advantage of TCR-NK in recognizing tumor cells with HLA I downregulation.

**Figure 2:**
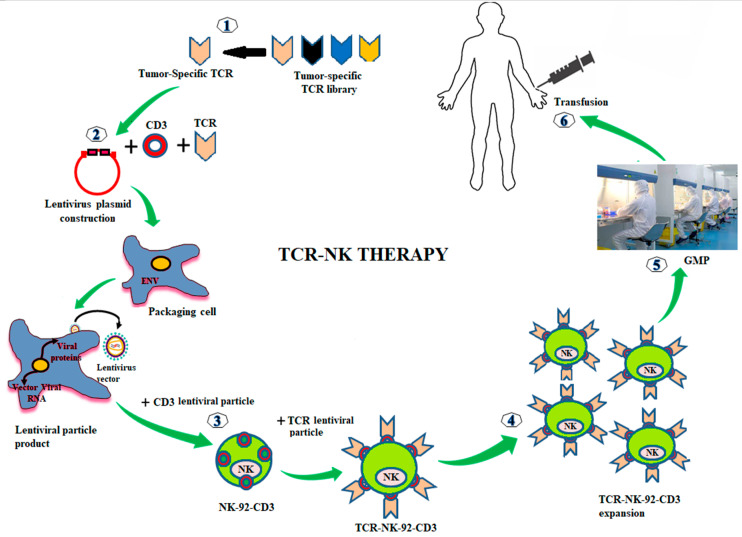
Genetic modifications of NK-92 cells with TCR for cancer immunotherapy. Tumor-specific TCRs are generated from the tumor-specific TCR library. TCRs are constructed into a lentivirus vector. The lentiviral particles of TCR are produced by transfection with the TCRs lentivirus vector into packing cells (293T cell). To make a stable expression of TCR and CD3 in NK-92 cells, the NK-92 cells are transduced with the lentiviral particles of CD3 (NK-92-CD3). Then, the stable expression of NK-92-CD3 is transduced with lentiviral particles of TCR (TCR-NK-92-CD3, in the short term called TCR-NK). The positive TCRs expressing in NK-92 cells are expanded ex vivo. TCR-NK products are manufactured under GMP-compliant conditions (e.g., irradiated cells prior infusion to patients). The final products of TCR-NK are infused into patients.

A schematic presents the techniques for the preparation of TCR-NK product is shown in Figure 2. Tumor-specific TCRs (e.g., NY-ESO-1 gene) and the four subsets of the CD3 gene were constructed into a lentivirus vector. Lentiviral particles can produce by transfection of the viral vector containing the TCR/CD3 gene into the package cells (e.g., 293 T cell). For producing TCR and CD3 stable expression in NK cell line (e.g., NK-92), the NK-92 cells were transduced with the lentiviral particles of CD3 (NK-92-CD3). NK-92-CD3 clone can achieve by shorting and seeding in 96 well-plaque. NK-92-CD3 cells were transduced with lentiviral particles of the tumor-specific TCR (TCR-NK-92-CD3, we called TCR-NK). The positive TCR-NK were shorted and seeded in 96 well-plaque to obtain the pure products. The TCR-NK products have tested the effect against the target cells and manufactured under GMP-compliant conditions. Therefore, the final TCR-products could be frozen and preserved in a therapeutic biobank, and they would be readily available for transfusion to patients.

Currently, two independent studies have shown that the transduction of TCRs along with all CD3 subunits redirected NK cell lines were specifically recognized tumor cells expressing the relevant antigen [15,16]. Parlar et al. [16] showed that TCR-NK (origin of NK-92 and YTS cell lines) against the HLA-A2-restricted tyrosinase-derived melanoma epitope (Tyr368-377) is MHC-restricted with the antigen-specific killing of tumor cells both in vitro and in vivo. Mensali et al. [15] also have reported the functional activities of TCR-NK (origin of NK-92 cell) against a TGF-βRII frameshift mutation peptide (TGFβRII131–139) and the melanoma-associated antigen melan-A peptide (Melan-A26-35) with a similar approach using all four CD3 chains. TCR-NK demonstrated enhanced antigen-specific recognition of target cells. The results from these pre-clinical studies showed the potential therapeutic efficacy of TCR-NK (Table 3).

As compared to other sources of universal T cells, the TCR-NK line can be genetically modified and expanded in vitro with less effort; furthermore, a pure proportion of TCR-NK products is much safer for clinical usage. TCR-NK can replace the high-cost TCR-T, which becomes effectively personalized for cancer immunotherapy. Moreover, without the expression of endogenous α/β TCR, infusion TCR-NK can be safe from severe toxicities, poorly immunogenic, or rejected by the host. Therefore, TCR-NK-based therapy might be more efficient for patients who failed several rounds of therapies, and might not qualify for autologous treatment conditions due to the poor quality of their immune cells

## 4. Conclusions

Despite the promising results of non-genetic- and genetic modified NK cells, NK cell-based cancer immunotherapy still has several challenges to overcome. Adoptive transfer of autologous-and allogeneic NK cells is hard to succeed in clinics due to the limitation of the number of infused cells. The optimization protocols may need to boost the number of the cell population for clinic usage. Although NK cell lines are unlimited cell expansions, which can provide benefit for cancer immunotherapy. The use of NK cell lines in the clinic has been demonstrated in low efficacy, and the lines needed to be irradiated in prior infusion to the patients. Even though NK cell-based monoclonal antibodies have been shown promising antitumor activity in patients with diverse tumor types, dose-limiting toxicities may need to manage and improve for the patient safety profile. Moreover, it remains some barriers for CAR-NK to accomplish in clinics, including the efficiency of CAR-transduction, limitation of cell expansion, lack of available targets, and weak elimination of the solid tumor. Therefore, some efforts should create to boost the efficiency of CAR-NK products. For TCR-NK-based therapy, many further studies must be continuing to investigate for the clinical application of TCR-NK in gene therapy, including engineering TCRs with primary NK cells or engineering NK cells with a variety range of TCR affinities for clinical benefit.

## Figures and Tables

**Table 1:** Strategies to eliminate tumors and some limitations.

Therapy	Strategies	Advantages	Disadvantages	References
Autologous NK cells	Systemic administration cytokines (IL)-2, IL-15, IL-18, IL-21, and interferon (IFN)α	Safe and widely used in clinic	Low efficacy caused by the suppression of recognition MHC molecule, cytokine administration	[33,34,35,36,37]
Allogeneic NK cells	In combing with interleukine (IL)-2, IL-12, IL-15, IL-18, IL-21 and IFN-α	Highly effective against KIR-ligand mismatch malignancies	Rejection by patient’s immune system, lack of antigen specificity, insufficient numbers	[38,39,40,41,42,43,44]
NK cell lines	Stimulated with cytokine IL-2, IL-12, IL-15, IL-18	Unlimited cell expansion, easily manipulated, high cytotoxicity, low cost	Low efficacy (except ha-NK), irradiated prior to clinical used,	[45,46,47,48]
Antibody-based NK cell therapy	Combined with mAb (e.g., cetuximab, rituximab, alemtuzumab, dinituximab)	More effective against cancers, higher cytotoxicity to Ab-coated target cells	Dose-related safety concerns	[49,50,51,52,53,54]
Genetic modification of NK cells	CAR-NK	Highly efficacy, stronger intracellular signals for activating NK cell cytotoxicity	Limited large-scale expansion of primary CAR-NK, low transduction efficiency, loss functional activity (freeze-thaw process), lack of available targets.	[9,11,55,56,57,58]
	TCR-NK (NK cell line)	Highly efficacy and safety, cost-effective, easily manipulated	MHC restriction	[15,16]

**Table 2:** Clinical study of CAR-NK in hematological and solid tumors.

NCT Number	Title	Conditions	Interventions	NK Source	Phase	Status	Locations
NCT03940833	Clinical research of adoptive BCMA CAR-NK cells on relapse/refractory MM	Multiple myeloma	Biological: BCMA CAR-NK 92 cells	NK-92	Phase 1 Phase 2	Recruiting	Department of Hematology, Wuxi People’s Hospital, Nanjing Medical University Wuxi, Jiangsu, China
NCT03940820	Clinical research of ROBO1 specific CAR-NK cells on patients with solid tumors	Solid tumor	Biological: ROBO1 CAR-NK cells	PB NK	Phase 1 Phase 2	Recruiting	Radiation Therapy Department, Suzhou Cancer Center, Suzhou Hospital Affiliated to Nanjing Medical University Suzhou, Jiangsu, China
NCT03692637	Study of anti-Mesothelin Car NK cells in epithelial ovarian cancer	Epithelial ovarian cancer	Biological: Anti-mesothelin Car NK cells	PB NK	Early phase 1	Not yet recruiting	Unknown
NCT03692663	Study of anti-PSMA CAR NK cell in castration-resistant prostate cancer	Castration-resistant prostate cancer	Biological: anti-PSMA CAR NK cells	PB NK	Early phase 1	Not yet recruiting	Unknown
NCT03692767	Study of anti-CD22 CAR NK cells in relapsed and refractory B cell lymphoma	Refractory B-cell lymphoma	Biological: Anti-CD22 CAR NK cells	PB NK	Early phase 1	Not yet recruiting	Unknown
NCT03690310	Study of anti-CD19 CAR NK cells in relapsed and refractory B cell lymphoma	Refractory B-cell lymphoma	Biological: Anti-CD19 CAR NK cells	PB NK	Early phase 1	Not yet recruiting	Unknown
NCT03415100	Pilot study of NKG2D-ligand targeted CAR-NK cells in patients with metastatic solid tumors	Solid tumors	Biological: CAR-NK cells targeting NKG2D ligands	PB NK	Phase 1	Unknown	Third Affiliated Hospital of Guangzhou Medical University Guangzhou, Guangdong, China
NCT03824964	Study of anti-CD19/CD22 CAR NK cells in relapsed and refractory B cell lymphoma	Refractory B-cell lymphoma	Biological: Anti-CD19/ CD22 CAR NK cells	PB NK	Early phase 1	Not yet recruiting	Unknown
NCT03579927	CAR.CD19-CD28-zeta-2A-iCasp9-IL15-transduced cord blood NK cells, high-dose chemotherapy, and stem cell transplant in treating participants with B-cell lymphoma	CD19 positive, B-cell lymphoma	Biological: Autologous hematopoietic stem cell transplantation, high-dose chemotherapy	CB NK	Phase 1 Phase 2	Withdrawn	M D Anderson Cancer Center Houston, Texas, United States
NCT02944162	CAR-pNK cell immunotherapy for relapsed/refractory CD33+ AML	Leukemia	Biological: anti-CD33 CAR-NK cells	NK-92	Phase 1 Phase 2	Unknown	PersonGen BioTherapeutics (Suzhou) Co., Ltd. Suzhou, Jiangsu, China
NCT04324996	A phase I/II study of universal off-the-shelf NKG2D-ACE2 CAR-NK cells for therapy of COVID-19	COVID-19	Biological: NK cells, IL15-NK cells, NKG2D CAR-NK cells, ACE2 CAR-NK cells, NKG2D-ACE2 CAR-NK cells	PB-NK	Phase 1 Phase 2	Recruiting	Chongqing Public Health Medical Center Chongqing, China

**Table 3:** TCR-NK cell-based therapy in pre-clinical study.

TCRs Used in the Study	Source of NK Cells	Diseases	Antigen-Specific Targets	Functional Activities	References
Tyr TCR	NK-92, YTS	Melanoma	HLA-A2/Tyr_368-377_ peptide (YMDGTMSQV)	Highly cytotoxicity and cytokine secretion with antigen-specific recognition both in vitro and in vivo	[16]
Radium-1 TCR and DMF-5 TCR	NK-92	Colorectal carcinoma and mantle B cell lymphoma	HLA-A2/TGFβRII_131-139_ peptide (RLSSCVPVA) and HLA-A2/Melan-A_26–35_ peptide (EAAGIGILTV)	Enhanced antigen-specific recognition of target cells both in vitro and in vivo	[15]

## Data Availability

No new data were created or analyzed in this study. Data sharing is not applicable to this article.
